# Exploring the Impact of Obesity on Skeletal Muscle Function in Older Age

**DOI:** 10.3389/fnut.2020.569904

**Published:** 2020-12-01

**Authors:** Paul T. Morgan, Benoit Smeuninx, Leigh Breen

**Affiliations:** ^1^School of Sport, Exercise and Rehabilitation Sciences, University of Birmingham, Birmingham, United Kingdom; ^2^Cellular & Molecular Metabolism Laboratory, Monash Institute of Pharmacological Sciences, Monash University, Parkville, VIC, Australia

**Keywords:** sarcopenic-obesity, muscle function, metabolic syndrome, sarcopenia, obesity paradox, anabolic resistance, intramuscular lipids

## Abstract

Sarcopenia is of important clinical relevance for loss of independence in older adults. The prevalence of obesity in combination with sarcopenia (“sarcopenic-obesity”) is increasing at a rapid rate. However, whilst the development of sarcopenia is understood to be multi-factorial and harmful to health, the role of obesity from a protective and damaging perspective on skeletal muscle in aging, is poorly understood. Specifically, the presence of obesity in older age may be accompanied by a greater volume of skeletal muscle mass in weight-bearing muscles compared with lean older individuals, despite impaired physical function and resistance to anabolic stimuli. Collectively, these findings support a potential *paradox* in which obesity may protect skeletal muscle mass in older age. One explanation for these paradoxical findings may be that the anabolic response to weight-bearing activity could be greater in obese vs. lean older individuals due to a larger mechanical stimulus, compensating for the heightened muscle anabolic resistance. However, it is likely that there is a complex interplay between muscle, adipose, and external influences in the aging process that are ultimately harmful to health in the long-term. This narrative briefly explores *some* of the potential mechanisms regulating changes in skeletal muscle mass and function in aging combined with obesity and the interplay with sarcopenia, with a particular focus on muscle morphology and the regulation of muscle proteostasis. In addition, whilst highly complex, we attempt to provide an updated summary for the role of obesity from a protective and damaging perspective on muscle mass and function in older age. We conclude with a brief discussion on treatment of sarcopenia and obesity and a summary of future directions for this research field.

## Introduction

The degenerative, generalized, and precipitous loss of skeletal muscle mass, quality and strength associated with aging is termed “sarcopenia” and is characterized by muscle loss of ~0.5–1.0% per year (1). The consequences of muscle mass and strength loss with aging are well-documented and encompass a number of physiological and non-physiological outcomes (1–7). In addition, sarcopenia is associated with decreased mobility and impaired whole-body metabolic health, as well as impaired function during locomotion, reduced resting energy expenditure, a reduction in non-structured free-living physical activity and increased fat mass (8, 9), factors that have also been found to be present with obesity. Evidence now exists to implicate the progressive deterioration of muscle quality, defined as muscle strength relative to a given quantity of muscle mass, to functional impairment with aging (2, 10, 11), including reductions in fiber size, number, and contractile function (12, 13), the degree of lipid infiltration (14, 15), and impaired neurological modulation of contraction (16). However, whilst worldwide rates of obesity have reached an all-time high (World Health Organization, WHO) and is associated with a plethora of comorbidities (17), research on how obesity might affect the progression of sarcopenia is in its relative infancy. Therefore, the purpose of this review is to explicitly focus on skeletal muscle deterioration in the presence of excess adiposity in older age and will explore some of the potential underlying mechanisms regulating changes in skeletal muscle function, with a particular focus on *in-vivo* models. The reader should be directed to recent reviews elsewhere for in-depth critical discussions on the potential multi-faceted causes of sarcopenia, obesity, and the confluence of these two conditions (7, 18–21). Whilst not a direct focus of the present review, the effects of obesity on molecular signaling associated with skeletal muscle contraction, bone health and the contribution of specific genetic and gut microbiome fingerprints on obesity as well to muscle mass/loss and its possible pleiotropic effects on adipose tissue are also reviewed elsewhere (22–25).

### Causes of Sarcopenia and Obesity

It is now widely acknowledged that the development of sarcopenia and loss of muscle strength is caused by the complex interplay of multiple factors beyond muscle atrophy (10, 26, 27) including; declines in neural function, hormonal changes, chronic low-grade inflammation, mitochondrial dysfunction, impaired/reduced satellite cell function, and lifestyle factors [e.g., malnutrition and physical inactivity; (2, 7, 21, 28–31)]. Similar to sarcopenia, the presence of obesity is also typically accelerated by chronic inactivity. Obesity is primarily caused by a progressive decline in total energy expenditure (i.e., decreased physical activity and reduced basal metabolic rate) in the presence of excessive caloric intake (20, 32). As opposed to the traditional model of sarcopenia, the high energy intakes observed with obesity may offer some protection against sarcopenic development by ensuring sufficient protein intake and supporting elevated muscle protein synthesis (20, 32–37). Growing evidence supports an underlying genetic component to the development of obesity (38–40). Whilst the major factors involved in obesity seem to be linked with dietary and physical activity habits, these factors have also been associated with the genome that may, independently, influence energy expenditure, fuel metabolism, muscle fiber function, gut microbiome, and hormone/appetite regulation (i.e., ghrelin and leptin) and/or food preferences (38–40).

### Health Consequences of Obesity

As aforementioned, alongside an aging population, levels of global obesity are on a progressive rise, with >30% of men and >40% of women currently classified as being overweight, and >10% as obese (41). An increase in whole-body adiposity is typically accompanied by a concomitant increase in ectopic fat deposition within skeletal muscle, termed myosteatosis (42, 43), both of which are associated with a plethora of comorbidities (17), including the progression of sarcopenia (44–47) and physical disability (48, 49). Independent of the effects of obesity on skeletal muscle health, adiposity is also a strong risk factor for poor overall health, reduced functional capacity and quality of life in older age (50–52). Furthermore, obesity has well-known metabolic effects that can lead to significant health complications such as metabolic syndrome (defined by the existence of a cluster of conditions including hypertension, high blood glucose levels, high serum triglyceride levels), Type II diabetes, and an increased risk of cancer (53, 54). In addition, obesity is a strong risk factor for atherosclerosis and other cardiovascular complications (55), as well as for many other chronic and acute diseases involving end-stage organ failure and infection (54–57), leading to further acute complications and prolonged periods of hospitalization. Unsurprisingly, the prevalence of many of the aforementioned medical complications are strongly associated with obesity in older age (58). Obesity is known to accelerate the risk of developing cardiovascular disease by increasing circulating lipids and glucose as well as impairing lipid and glucose metabolism and increasing systolic and diastolic blood pressure (59–61). In addition, obesity is strongly associated with elevated chronic systemic inflammation and progressive declines in physical activity and cardiorespiratory fitness, which all contribute to a worse prognosis of ill health and accelerated sarcopenia risk (59–62). Obesity is associated with a number of endocrinological impairments (63). For example, in obese adipose tissue adipocytes undergo hypertrophy, hyperplasia and activation resulting in accumulation of pro-inflammatory macrophages and other immune cells as well as dysregulated production of various adipokines that together further exacerbate inflammation (23). The excessive production and impaired capacity to store lipids in old obese individuals is also known to increase reactive oxygen species and impair metabolic function that is ultimately capable of inducing metabolic dysfunction (notably, impaired mitochondrial and skeletal muscle function via elevated inflammation) (23). Whilst adiponectin, an adipokine produced primarily by adipose tissue, is known to hold a number of important metabolic functions (e.g., insulin-sensitizer and anti-inflammatory), these responses become dysregulated with obesity, making skeletal muscle particularly susceptible to metabolic impairments and increased risk of morbidity (64, 65). It has been suggested that a vicious circle of maintaining a high volume of adipose tissue and skeletal muscle inflammation, triggers the development and acceleration of sarcopenic-obesity with a complex interplay between a number of mechanisms that are beyond the scope of this review (i.e. adipocyte-like phenotype of muscle progenitor cells, insulin resistance, impaired neuromodulation of contraction, leptin resistance, impaired endocrine function, reduced release of adiponectin, increased glycation products and oxidative stress etc.) (23, 63, 66). However, less is known on how obesity, *per se*, might affect the progression of sarcopenia in older age and/or in pre-sarcopenic individuals. Undoubtedly though, myosteatosis has the potential to exert detrimental local effects on muscle contractility given its close proximity (67) and this is discussed below.

### Diagnosis and Prevalence of Sarcopenic-Obesity

The combined presence of sarcopenia and obesity (i.e., sarcopenic-obesity) is rapidly increasing in older adults. Changes to body composition, including reduced muscle mass concomitant with an increase in fat mass and simultaneous reduction in resting metabolic rate, are evident with aging (68). Whole-body muscle mass and strength start to decline progressively as early as 30 years of age, with a more accelerated loss after the age of ~60–70 years (69–71). The prevalence of metabolic syndrome also increases with age, with a rise after the 3rd decade of life, reaching a peak between 50 and 80 years (72, 73). Importantly, as skeletal muscle plays a critical role in glycaemic control and metabolic homeostasis (74, 75), the combination of a loss of lean body mass and a concomitant increase in visceral adiposity will likely exacerbate the risk of developing metabolic disease, particularly in the older adult, accelerating skeletal muscle deterioration, reduced physical performance, an increased risk for disability, hospitalizations, morbidity, and early mortality (76–78). The development of sarcopenic-obesity is thought to be brought about by a combination of factors that contribute to the development of both conditions (21) and is associated with significantly greater body fat, oxidative stress and inflammation and intracellular lipotoxicity, glucose and endocrine dysregulation, as well as impaired muscle strength and lower lean body mass (63, 66, 79–81). Further, when obesity and impaired muscle function co-exist, they act synergistically in a “vicious cycle” on the risk of developing multiple health-related outcomes (19, 21, 42, 82–84).

**Figure 1:**
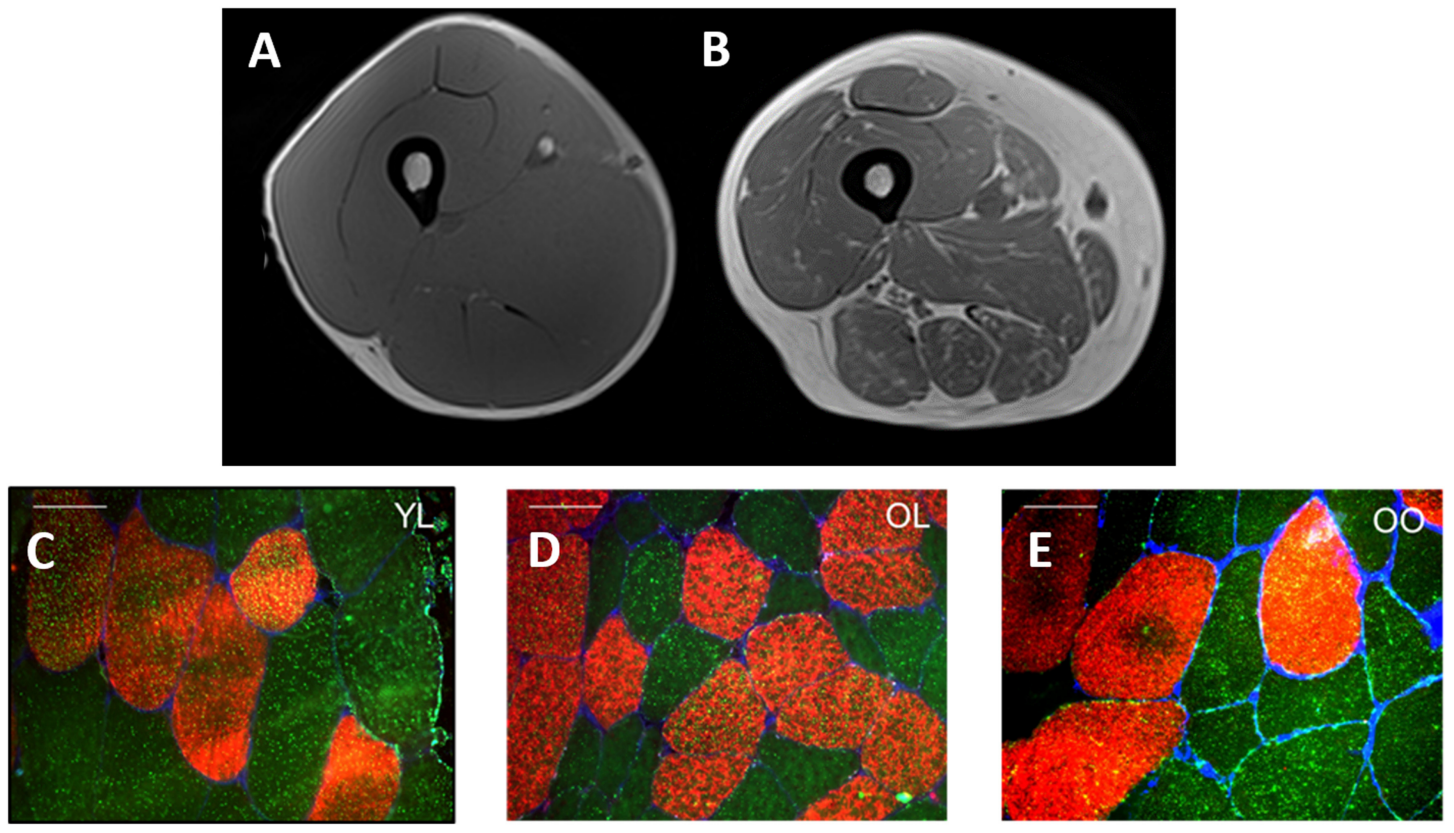
Cross-sectional MRI of the midpoint of quadriceps muscle from a young healthy individual **(A)** and pre-sarcopenic older individual **(B)** with a similar thigh circumference (~60–65 cm) and BMI (~26 kg·m^2^). The young-healthy individual exhibits a larger quantity of skeletal muscle mass **(A)** whereas high infiltration by adipose tissue is observed in the pre-sarcopenic older individual **(B)**. **(C–E)** Provides an illustration of representative fiber cross-sectional images stained for Myosin Heavy Chain I (red) and lipid droplets (bodipy, green) in young lean (YL), old lean (OL), and older obese (OO), respectively. Notably, type I fiber lipid droplet number is significantly greater in young lean (*n* = 616) compared with older lean (*n* = 412) and older obese (*n* = 533) and greater in olden obese compared with older lean (**C–E**, respectively) indicative of an ‘athlete paradox', whereby young, healthy individuals exhibit higher levels of intramuscular lipids but with the superior capacity for oxidation. In addition, type II fiber lipid droplet number and area is significantly greater in the young lean (*n* = 377) and old obese (*n* = 415) compared with old lean (*n* = 242) individuals, despite poor whole-body metabolic health in the former (i.e., old obese), indicative of poorer muscle quality and impaired oxidative function. Further, in this study, type I and II muscle fiber cross-sectional area was greater in young lean (4,031 ± 1,978 μm^2^, 4,009 ± 1,733 μm^2^, respectively) compared with older obese (3,421 ± 1,528 μm^2^, 3,390 ± 1,199 μm^2^, respectively) and older lean (3,009 ± 1,251 μm^2^, 2,170 ± 1,243 μm^2^, respectively) groups and significantly greater in old obese compared with older lean [from Smeuninx et al. (85)]. White bars for **(C–E)** represent 50 μm.

Multiple definitions exist for sarcopenic-obesity (21, 63), however, the use of gross metrics such as body mass and BMI, often provide an incomplete understanding of the person's actual body composition. This paradigm is depicted in Figure 1 which illustrates a cross-section of the thigh taken by magnetic resonance imagery (MRI) from a young healthy individual (Figure 1A) and pre-sarcopenic older individual (Figure 1B) with similar thigh circumferences (~60–65 cm) and similar BMI (~26 kg·m^2^). Despite a similar initial appearance, the differences in the composition of the muscle between individuals are clear. In the young-healthy individual, the portion of muscle mass is relatively large with minimal adiposity and lipid infiltration. In contrast, the pre-sarcopenic older tissue is significantly smaller and contains a much larger layer of subcutaneous fat and lipid infiltration within and between muscles of the thigh. In this example, the pre-sarcopenic individual presents with a relatively “normal” body mass and BMI but stores higher and relatively lower amounts of adipose and lean tissue, respectively. Therefore, the pre-sarcopenic individual presents a significantly elevated risk of developing metabolic complications. Figures 1C–E demonstrate a significant greater lipid droplet number and area in type II fibers of obese older compared with healthy young and healthy old individuals, which could be indicative of poorer muscle quality and impaired oxidative function in older obese individuals. The precise identification of sarcopenia and obesity is of vital importance for accurate diagnosis and treatment of sarcopenic-obesity (84). Importantly, as obese older adults typically experience a reduction in relative (i.e., %) muscle mass as opposed to a loss in absolute (i.e., kg) muscle mass (44), sarcopenia in obese individuals is often characterized based upon changes to muscle quality and/or relative rather than absolute levels of skeletal muscle mass (86–88). A number of tools are, therefore, used in combination to assess body composition (i.e., MRI, CT, DEXA, BIA), muscle strength (i.e., dynamometry, 1- and 12-repetition maximum testing, grip strength), and physical performance [i.e., gait speed, stair climb, chair rise time, standard physical performance battery tests (SPPB)]. The following section will discuss the potential implications of obesity for aging muscle, with a specific focus on muscle morphology and the existence of an “obesity-paradox.”

## Implications of Obesity for Aging Muscle

### Obesity and Muscle Morphology

It is well-recognized that aging is typically characterized by a slowing of muscle phenotype toward type I and a concomitant reduction in type II muscle fiber area and number [i.e., (89)]. However, in contrast to these typical symptoms of aging, obesity seems to be associated with a more predominant faster phenotype (particularly type IIx) and smaller percentage of type I muscle fibers (85, 90, 91), with a negative relationship between the degree of adiposity and the relative percentage of type I muscle fibers, comparable to that observed with reduced physical activity (92–95). In further support of this phenomenon, following a weight loss intervention, a positive relationship between the percentage of excess weight loss and the percentage of type I fibers has been found in morbidly obese individuals (91). Whilst there is evidence to suggest that a relative reduction in type I fibers is a result of excess adiposity, it is also possible that this may reflect an intrinsic defect predisposing individuals toward obesity (91). This is pertinent to note as, aside from any impact on functional outcomes, a relative reduction in type I muscle fibers is also related to impaired metabolic health, increased LDL content, decreased insulin sensitivity and decreased arterial elasticity (90). In contrast to type I muscle fibers, type II muscle fibers possess impaired lipid disposal capabilities, thus contributing to a reduction in whole-body lipid oxidation and an increased storage of lipids (96–98). Type II muscles fibers are more closely associated with higher oxidative stress which may, in turn, negatively impact the integrity of mitochondria and lead to an apoptotic cascade that may ultimately result in cell death (99). Others have also reported that skeletal muscle from obese individuals has markedly lower mitochondrial content and a concomitant lower oxidative capacity, which is also linked with insulin resistance (43, 96, 100–103). A recent review has found adiposity to have profound negative impacts on cellularity, secretory profiles, and inflammatory status, which drive lipotoxicity of skeletal muscle and negatively impact muscle fiber contractility (78). In contrast, an increase in the absolute number and size of type II muscle fibers is associated with indices of muscle strength and power, and, thus, may contribute to a reduced risk of falls and fracture which can have important implications for muscle loss in aging (104). However, both aging and obesity are independently associated with muscle atrophy and reduced myogenesis, as well as impaired excitation-contraction coupling (105, 106). Obese individuals are also known to possess impaired functional capacity (48, 107) and fat mass is negatively associated with jump height in middle-aged and older adults (108). An important consideration in these individuals is the significantly higher intramuscular lipid content in type II fiber (compared with older lean controls) combined with impaired muscle fiber contraction, lower relative muscle strength and impaired oxidative function, indicative of poorer muscle quality (85, 109–112). At least in old sarcopenic-obese rodents, ectopic fat deposition seems to also contribute to anabolic resistance (113). We have also previously shown type I fiber intramuscular lipid content to be equivalent between young lean and old obese, but not old lean, individuals (85) (Figure 1). However, type I fiber intramuscular lipid content was negatively associated with insulin sensitivity for old lean and old obese, but not young lean individuals (85), reinforcing the notion that the capacity for intramuscular lipid oxidation is likely greater in young lean and, in particular, significantly impaired in older obese individuals (14, 85). It is also pertinent to note that such observations are also likely to be related to aerobic fitness/physical activity levels rather than obesity, *per se*. Indeed, both impaired oxidative capacity and increased muscle IIx fiber number are both typical outcomes of physical inactivity (114). Nevertheless, as reduced physical activity is typically an inherent characteristic of obesity, it is often difficult to distinguish between the two. It is plausible that a physically active older obese individual might possess greater intramuscular lipid oxidation capabilities compared with a inactivity older obese individual (85).

Obesity is also characterized by the induction of skeletal muscle remodeling as well as mitochondria-mediated apoptosis (115). On the contrary, exercise has been reported as a positive regulator of skeletal muscle remodeling and apoptosis, however, as aforementioned, obese individuals typically present with low levels of physical activity (115). Interestingly, it has been suggested that the impaired function of mitochondria with obesity may be triggered by altering the expression of mitochondrial proteins regulating key metabolic processes in skeletal muscle likely due, in-part, to the subcellular localization of lipid droplets and a reduction in the amount of skeletal muscle perilipin 2 protein per intramyocellular lipid, particularly in type II muscle fibers (116, 117). A recent review has also demonstrated that such effects of obesity on muscle morphology and contractile performance may be muscle specific, such that obesity may increase the absolute force producing capacity of the postural and antigravity muscles (22, 118). Further, studies examining the contractile performance of isolated muscle suggest that obesity may accelerate the age-related decline of respiratory, but not locomotory weight-bearing, muscles (119). However, whilst a high-fat diet (and an associated increase in body and fat mass in rodents) may contribute to increased muscle mass and strength, the increase in absolute strength is smaller than the magnitude of weight gain, meaning that *in-vivo* locomotor function is likely to be impaired in old obese adults (119). In further support of this notion, a recent study demonstrated an additive effect of obesity, induced by a high-fat diet on the reduction in contractile function with aged rodents, demonstrating that the increase in intramyocellular lipid levels were associated with the degree of impaired muscle contractile force (120, 121).

### A Paradox of Obesity in Older Age?

Whilst many studies have shown that muscle strength and, to some extent, muscle mass are strong predictors of survival (2, 71, 122–124), the relationship between obesity and mortality remains equivocal (125–127). Studies have shown that obese individuals seemingly maintain a higher absolute quantity of muscle mass compared with their lean counterparts (85), supporting the existence of an aging “obesity paradox” (128). The existence of an obesity paradox has been observed in a range of populations including, for example, patients undergoing lung cancer surgery (129), with overall morbidity and in-hospital mortality significantly decreased in obese patients (129). Whilst a number of studies have shown a BMI in the “normal” range (18.5–25 kg·m^2^) to be associated with the lowest risk of mortality (130, 131), other large cohort studies have demonstrated a “survival benefit” for overweight (25–30 kg·m^2^) and even obese (≥30 kg·m^2^) individuals, suggesting a protective effect of obesity against mortality in older age (125, 132–134). By contrast, a recent study found that whilst waist circumference was associated with greater muscle size, this was also associated with impaired muscle function (assessed via leg extension strength, chair stands and stair climb time, gait speed, and SPPB scores) as well as with measures of muscle quality (135). It is widely accepted that, with increasing body mass, absolute skeletal muscle mass increases, and this might explain these consequential findings in obese individuals (87). In support of this notion, reduced survival for individuals with a ‘normal’ (18.5–25 kg·m^2^) or ‘low’ (≤18.5 kg·m^2^) BMI, compared with overweight (≥25 kg·m^2^), might be explained by loss of muscle mass in the former (136). A recent study found that skeletal muscle mass mediates associations of BMI with adiposity and mortality, such that irrespective of adiposity and BMI, muscle mass was inversely associated with the risk of mortality (137). Excluding participants with low muscle mass across all groups also alleviated the risk associated with a low BMI, but magnified the risk associated with a high BMI, such that survival was greatest with a normal BMI (137). These data are in agreement with a recent cohort study sampling >38,000 men with >12,000 deaths, suggesting that the obesity paradox is likely explained by low levels of lean mass, rather than low fat mass, in the lower range of BMI, such that a “J” shaped association was consistently observed between BMI and all-cause mortality (138). In contrast, a “U” shaped association was found between predicted lean body mass and all-cause mortality (138). Thus, more recent observations are not consistent with a survival advantage related to overweight or obesity, but rather to elevated levels (or better preservation) of muscle mass in aging and “at risk” groups. Individuals with a BMI of ≥30 kg·m^2^ have, indeed, been shown to be at a significantly higher risk of functional impairment (139).

**Figure 2:**
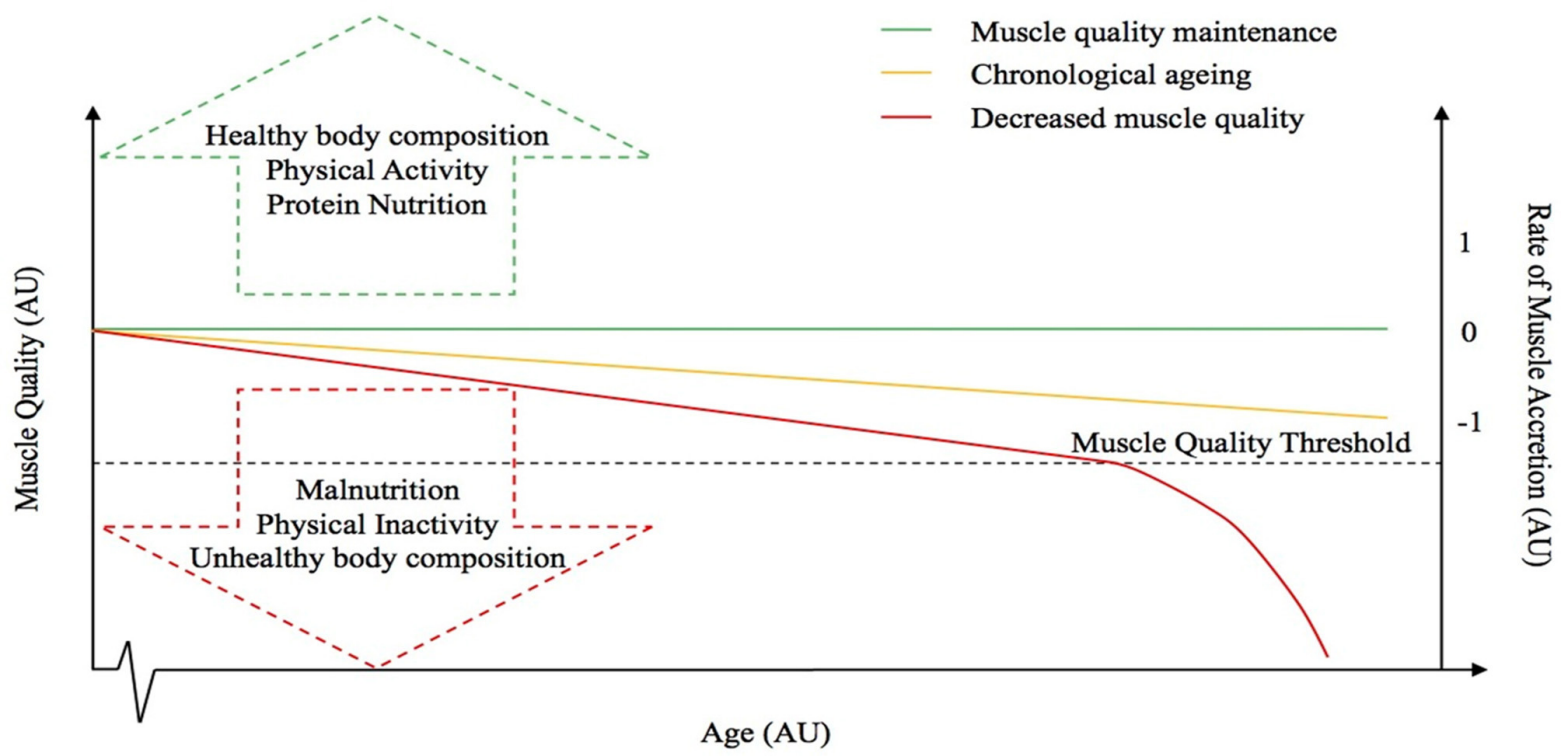
Hypothetical relationship between muscle quality and the rate of muscle accretion or loss with advancing age. The dotted line represents the “muscle quality threshold” beyond which obesity-induced muscle anabolic resistance results in a precipitous, rapid decline in muscle mass and function. The arrows represent some of the factors that might influence muscle quality. The red line represents a decrease in muscle quality, whereas the green line is representative of muscle quality maintenance and the yellow line depicts the “normal” chronological aging process.

The principle factors driving individuals toward either the sarcopenic-obese or healthy-obese phenotype are currently unknown but might include differences in physical activity and caloric intake as these are known regulators and associating factors to many of the potential causes of sarcopenia and obesity (i.e. reduced lean mass, elevated oxidative stress and inflammation, impaired muscle regeneration, anabolic and insulin resistance, nutrient ‘overload’ and metabolic dysfunction etc.) (66, 80). Furthermore, the inability to delineate sarcopenic-obesity based on BMI alone, adds to the misclassification of certain individuals (140). Indeed, whilst obese individuals are typically less physically active, observations of higher levels of muscle mass in obese groups are likely a response to an elevated overload stimulus due to the requirement to move an increased body mass. It is reasonable to speculate that the role of other factors, such as genetics, may also contribute to the discrepancies observed between these two aging groups. As aforementioned, whilst Smeuninx et al., amongst others, have shown that obese individuals seemingly maintain a higher absolute quantity of muscle mass, greater insulin resistance is also observed in these individuals compared with lean age-matched controls, suggesting that the quality, and not necessarily quantity, of skeletal muscle may play a more relevant role in whole-body metabolic health, particularly in aging (85). Observations to date question the role of obesity to skeletal muscle anabolism, and, interestingly, whether a “threshold” exists after which obesity becomes deleterious. Based on our observations, and those of others, we postulate the existence of an aging-obesity “muscle quality threshold” beyond which, obesity-induced muscle anabolic resistance results in a precipitous, rapid decline in muscle mass and function (Figure 2), centered on muscle quality rather than absolute levels of skeletal muscle mass. Research suggests that obesity may have differential effects on muscle metabolism and preservation of muscle mass depending on age (88) and it is likely that being young, physically active and not suffering from the longer-term impacts of obesity, may also mediate some level of protection against any obesity-related anabolic resistance (141). Thus, obesity may not be problematic for muscle mass and function unless present under situations of aging, chronic inactivity, and/or other known risk factors for anabolic resistance (142–147). Undoubtedly, more studies are required to examine the metabolic, morphological, and functional characteristics related to obesity, particularly in pre-sarcopenic individuals and/or in the earlier stages of the aging process to better understand the principle factors driving individuals toward either the sarcopenic-obese or ‘healthy’-obese phenotype.

## Dysregulation of Skeletal Muscle in Obesity and Aging

### Regulation of Muscle Proteostasis

The regulation of skeletal muscle mass is a complex process that involves the precise coordination of several metabolic and intracellular signaling pathways, ultimately affecting the dynamic balance between muscle protein synthesis (MPS) and muscle protein breakdown (MPB). In order to increase skeletal muscle mass, MPS must exceed MPB, which can be achieved by performing regular resistance exercise training in combination with adequate protein nutrition (148–151). By contrast, dysregulation of MPB in combination with impaired and/or normal MPS is observed in numerous conditions such as aging and prolonged immobilization (152, 153). In a young, healthy individual, skeletal muscle turnover is ~1–2% per day (154). Indeed, following the intake of as little as ~10 g of dietary protein, a transient robust increase in MPS is observed (155–158) and with the addition of exercise, the duration and the magnitude of this anabolic response can be potentiated (158–160). Whilst the addition of regular physical activity elevates MPB, when physical activity is combined with sufficient protein intake in healthy individuals, the outcome will be a favorable elevation in MPS and net protein accretion (155). Indeed, physical activity is an important locus of control in the regulation of skeletal muscle mass in both young and older individuals, with increased physical activity levels stimulating MPS (161) and decreased physical activity levels blunting MPS (142).

### Old Age and Muscle Proteostasis

Whilst young individuals demonstrate a pronounced response to anabolic stimuli, a blunted response has been observed in older adults (157, 161–163). These studies amongst others, indicate the importance of increasing daily, per meal and quality of protein doses as well as the intensity and volume of exercise to restore muscle anabolic sensitivity in the older adult (164–167). Recent studies have also demonstrated anabolic resistance (defined as a diminished MPS response to food intake and exercise) in older adults following a chronic exercise training program (168), which suggests that by extension, aspects of chronological aging are inevitable, and that regular physical activity becomes a less effective stimulus for muscle remodeling and maintenance. It has been suggested that the post-prandial inhibition of MPB may also be impaired in older adults in combination with a blunted post-prandial MPS stimulation (169). This age-related anabolic resistance is likely an important driving factor in age-related muscle loss and appears to be exacerbated by aspects of biological aging such as intermittent periods of musculoskeletal disuse and reduced physical activity (87, 104, 142, 144, 145, 157, 165, 170, 171). Aging is also associated with impaired molecular regulation of skeletal muscle signaling pathways. Growing evidence suggests that mTOR complex 1 [mTORC1] (mammalian target of rapamycin) signaling, which is thought to have a regulating impact on protein synthesis, influences the aging process as acute inhibition of this pathway (by rapamycin) seems to extend the lifespan in model organisms and provides protection against a number of age-related pathologies (172–177). In addition to impaired mTORC1 signaling, impaired satellite cell proliferation is associated with aging (99, 178). This has important implications in the aging adult as satellite cells are thought to play a critical role in muscle fiber growth, muscle tissue turnover, and regeneration and are activated and proliferate in response to anabolic stimuli (179). Changes in the secretion of sex-hormones (i.e., growth hormone, testosterone, estrogen) are also associated with chronological aging (180). There has also been a recent interest in the role of a number of other factors involved in an individuals' dietary status, beyond protein intake, that have been implicated in the age-associated deterioration of skeletal muscle mass. For example, whilst the role of vitamin D status in sarcopenia is not well-understood, it has been suggested that older adults are at higher risk of lower levels of vitamin D as a result of decreased cutaneous synthesis and dietary intake of vitamin D (181, 182) and epidemiological evidence indicates an association between low levels of vitamin D and diseases associated with aging (182). Specifically, vitamin D induces myogenic differentiation in skeletal muscle derived stem cells (183). Similarly, there has also been a recent rise in the interest of the role of vitamin D receptors in the nervous, cardiovascular, and endocrine systems that may interact to accelerate sarcopenia progression (184, 185).

### Obesity and Muscle Proteostasis

**Figure 3:**
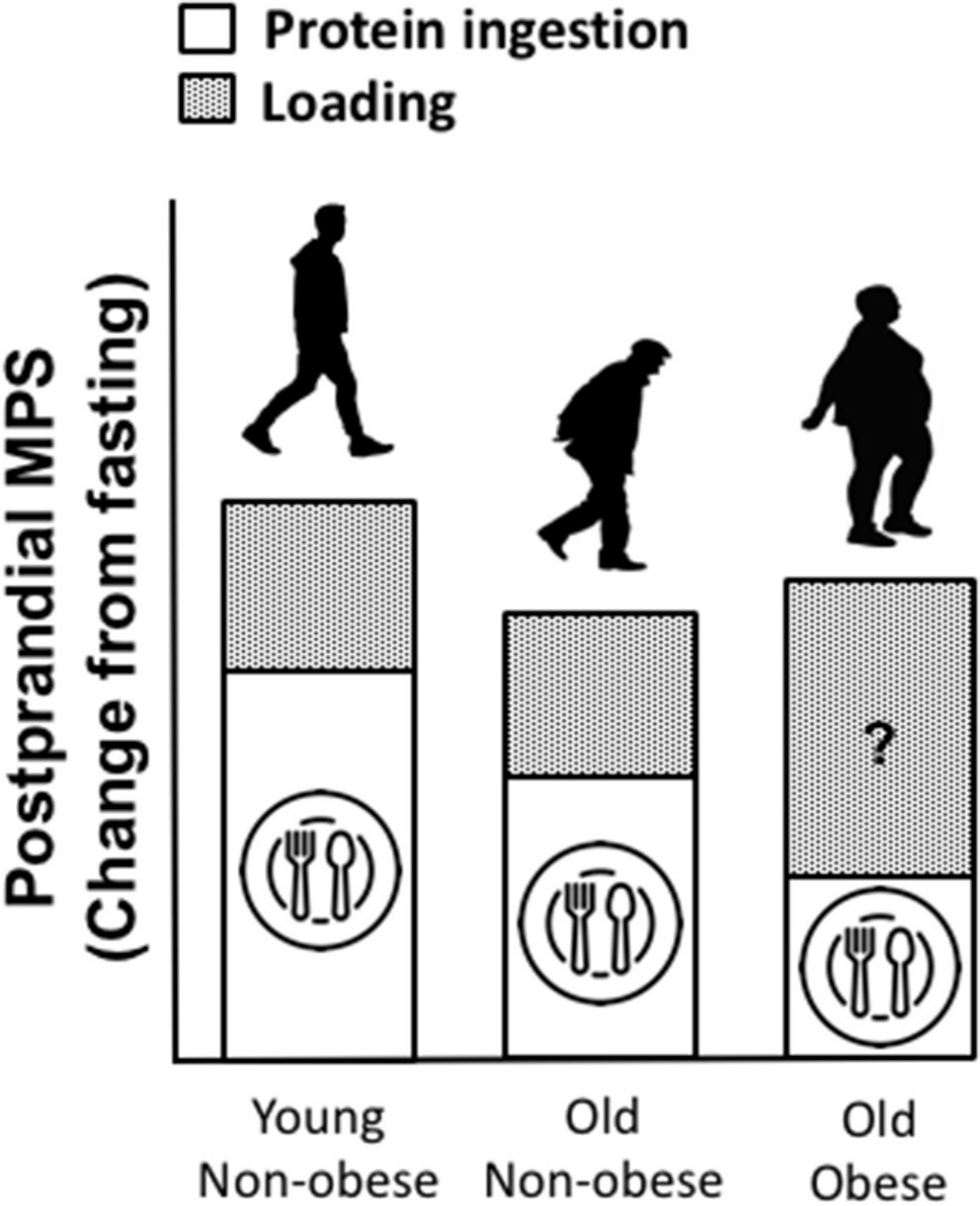
Proposed impacts of obesity on muscle protein turnover (MPT), in a fed state (post-prandial) and following a load-bearing activity (assuming sufficient protein intake). We speculate that, as lean mass is often preserved in obese vs. lean individuals and as obese individuals are subjected to greater loading forces and increased contractile work during activity due to the required to move more inert mass, this might elicit a positive training effect, driving muscle protein synthesis (MPS) following periods of movement, compensating for diminished MPS in response to protein provision. However, whilst little is known of the impact of obesity on MPB, there is evidence to suggest that MPB may be elevated due to a higher inflammatory burden associated with obesity.

As aforementioned, infiltration of fat is evident within muscle (termed “myosteatosis”) and this typically increases with age. Similar to chronological aging, it is generally agreed that rates of basal MPS are similar between obese and lean individuals (186). However, muscle anabolic resistance is exacerbated with obesity and thus the combination of obesity with older age leads to a near non-existent elevation of the MPS response to amino acid provision beyond basal levels, which may be associated with a plethora of factors including; reduced physical activity and a low relative proportion of lean mass, intracellular lipotoxicity and elevated inflammation, impaired muscle regeneration, insulin resistance, impaired endocrine function, impaired anabolic signaling etc. (85, 187, 188). Whilst little is known of the impact of obesity on MPB, there is evidence to suggest that MPB may be elevated due to a higher inflammatory burden associated with excessive adiposity and overfeeding (188–199). It has been suggested that the muscle anabolic resistance observed in obese individuals may be underpinned by impaired/inhibited muscle anabolic signaling phosphorylation (200, 201). Taken together, the available data suggest the possibility of an obesity-induced inability to regulate muscle protein turnover in response to nutrition, which may lead to impaired skeletal muscle remodeling. In contrast to suggestions of elevated MPB, the impairment of MPS following protein nutrition in obese may be offset by inhibition of MPB, implicating a possible protective mechanism to explain the apparent preservation of muscle mass in obese vs. lean older adults (188). It is also worthy of note that, as obese individuals are subjected to greater loading forces and increased muscle contractile work during activities of daily living (due to the requirement of moving more inert mass), this might elicit elevations in MPS and offer a degree of “protection” against the loss age-related muscle mass loss [(85, 187, 188, 202, 203); Figure 3]. Indeed, there is currently not a clear consensus for the impacts of obesity on MPS. There is no obvious explanation for the discrepancies observed between studies on the impacts of obesity on post-prandial MPS (85, 186–188, 204, 205). However, differences in habitual levels of physical activity as well as the immediate effects of exercise on indices of skeletal muscle anabolism, might explain in part, some of these contrasting observations between lean and obese individuals. Indeed, we showed that the MPS response to protein ingestion correlated with physical activity (as assessed via daily step count, *r* = 0.57) (85). Further, some of these discrepant findings might be explained by the assessment of MPS under hyperinsulinemic-hyperaminoacidemic conditions, which are not representative of a normal physiological response (186, 187). Anabolic resistance to protein provision has also be associated with obesity due to overfeeding and/or lipid oversupply (187, 188, 204), and therefore, the predominant cause(s) of obesity should also be considered. Nonetheless, irrespective of these discrepant findings, the magnitude of any potential “training” effect of physical activity, as discussed above, is typically lower than the increase in body weight observed with excess adiposity and is likely insufficient in offsetting sarcopenia (119, 206).

Whilst high-fat diets, lipid administration, obesity, and ectopic fat deposition have all been shown to induce whole-body and muscle anabolic resistance (113, 187, 204, 207, 208), as aforementioned, data also implicates obesity in impairing MPS through compromised muscle quality and/or elevated levels of adipose-derived inflammatory cytokines (139, 209). Indeed, a number of lipid species and metabolites have been implicated in impairing muscle function as well as developing anabolic resistance (210–216). However, despite previously observing a negative association between the net post-prandial MPS response following ingestion of a moderate protein dose (15 g milk protein) and leg fat mass, at least in our hands, we were unable to demonstrate any association with muscle fiber-specific intramuscular lipid content (85). This was despite a two-fold higher type II fiber intramuscular lipid content in older obese compared with older lean and young lean individuals. This, however, is in contrast to previous reports of associations between intramuscular lipids and muscle anabolic resistance in old rodents (113). Type I fiber intramuscular lipid content was, though, negatively associated with insulin sensitivity for old lean and old obese, but not young lean. This reinforces the notion that the capacity for intramuscular lipid oxidation is likely greater in young lean individuals and that this may contribute to metabolic resistance in the former (14). Activity-matched older lean and older obese comparisons are required to confirm such speculation (14). The subcellular characteristics, location of intramuscular and/or specific class of lipid intermediates (i.e., ceramides, diacylglycerol, etc.) may associate more closely with age-related muscle anabolic resistance (217). The specific role of perilipins, a lipid droplet-associated protein, requires further research (213, 215), particularly as there is lack of research investigating anabolic resistance in response to load carriage work in obese older vs. lean age-matched individuals. Taken together, these data may support an obesity-induced blunting of post-prandial MPS due to intramuscular lipid accumulation. We speculate that any potential protection of adiposity on skeletal muscle mass is likely solely driven by physical activity and that decreases in muscle mass and function in individuals diagnosed with sarcopenia and obesity combined are likely to be accelerating at a rate whereby levels of adiposity fail to provide any protection, but rather compound disease progression. Importantly, and as aforementioned, the causes of obesity and muscle loss are likely interrelated and interact. For example, research suggests that increasing storage of fat may also influence sedentary behavior, producing a vicious cycle of skeletal muscle deterioration and impaired whole-body metabolic health (218). Therefore, appropriate treatment for obesity (and sarcopenic-obesity) is imperative in improving muscle function and quality of life in older age.

## Treatment of Sarcopenia and Obesity

Treating obesity appropriately in older age is of upmost importance in order to prevent any further loss of skeletal muscle associated with aging (219–221). Treatment of obesity in older age (and sarcopenic-obesity) requires a combination of personalized nutrition and physical activity approaches as, under certain conditions, inappropriate changes to these paradigms may actually accelerate the progression of sarcopenia given the susceptibility to energy restriction and, thus, a potential catabolic state (222). As the pathogenesis of these three conditions (sarcopenia, obesity, and sarcopenic-obesity) is multi-faceted, understanding the optimal treatment is highly complex. Optimal treatment of sarcopenic-obesity likely incorporates a combination of resistance and aerobic training in the presence of a small dietary calorie deficit with sufficient protein intake, as severe weight reduction strategies may also compromise the ability to preserve muscle function and mass which may subsequently lead to frailty, disability, and increased morbidity and mortality (220, 223). It is also noteworthy that bone density may also be impaired with weight-loss programs which may, in-turn, contribute to fracture risk, and subsequent further risk of hospitalization, periods of prolonged inactivity and further muscle loss and functional impairment (87, 104, 222). Nevertheless, weight loss should be considered safe in older adults, if managed appropriately (222, 223). As such, treatment of sarcopenia alone may be easier than treatment of (pre)-sarcopenic obese individuals. Accumulating evidence also indicates that diet-induced weight loss can improve physical function among obese older adults, independent of any changes in physical activity (224–226). Incorporation of resistance exercise, in particular, into a training program is extremely important in reversing the symptoms of obesity in older age. Studies suggest that when a diet and exercise intervention is administered to obese individuals, subsequent improvements in muscle strength and muscle quality, are observed (227, 228). More recent attempts to assess the effectiveness of chronic training paradigms in obese older individuals have investigated the potential of a combined aerobic and resistance training approach and found positive outcomes when combined to either alone (229). Resistance exercise is known to have a number of effects that have the potential to prevent and/or overcome anabolic resistance including: enhanced post-prandial muscle protein synthesis, sensitized muscle insulin action, increased number and size of muscle fibers (particularly fast twitch) and reduced inflammation [i.e., IL-6 and TNF-a; (160, 230, 231)]. Aerobic exercise has also been shown to improve oxidative capacity, reduce intramyocellular lipid accumulation, enhance lipolysis and improve glucose utilization and insulin sensitivity (232), all of which have the potential to improve symptoms of sarcopenia and/or obesity in older age. Whilst we recognize that research suggests that weight loss in obese individuals is also typically followed by a further period of weight gain (233–236), it is important to note that the metabolic health benefits of weight loss and increased physical activity are likely sustained for a prolonged period (234, 235). Indeed, even when weight has been regained, long lasting benefits of weight loss are observed on a number of important metrics including insulin sensitivity (237). Accordingly, a multi-faceted approach to the prevention, management and treatment of sarcopenic-obesity remains the most promising.

Taken together, as undoubtedly older individuals with higher amounts of muscle mass are less likely to suffer from the inevitable impacts of age-related muscle loss, it would be prudent to increase basal levels of muscle mass, preferably, via a well-programmed resistance exercise training programme possibly combined with small increases in fat mass and body weight. It is, however, important to recognize that excess weight gain via increases in fat mass, whilst may be associated with higher volumes of muscle, are likely to impair muscle function and accelerate the age-related impairment in muscle function by significantly impairing muscle quality. In contrast, given the potential for muscle mass in obese older adults to be somewhat protected from sarcopenia, it *may* be prudent to suggest individuals carry a small amount of extra weight in older age, at a consequence of a small sacrifice in muscle quality, in support of a “muscle quality threshold” (Figure 2). Whilst speculative at this stage, this may help achieve a delicate balance of increasing lean and fat mass without the deliberating effects of excess adiposity. However, whilst the optimal strategy in pre-sarcopenic individuals is unclear, older individuals who are under weight and seemingly at an elevated risk, may benefit from small increases in fat mass. Undoubtedly though, promotion of a healthy lifestyle for chronic prevention (or attenuation), rather than treatment, *per se*, of sarcopenia, obesity, and sarcopenic-obesity should be considered the *best* approach by promoting adaptations of higher basal levels of muscle mass. Therefore, a combination of progression physical activity and gradual well-planned caloric restriction are likely key to achieving improved health outcomes in overweight individuals (84, 219, 238, 239). For more comprehensive reviews on the treatment of sarcopenia, obesity and sarcopenic-obesity in older age, the reader is directed to the following [i.e., (216, 232, 233)].

## Conclusions

### Future Directions

Throughout this review, we have provided some suggestions on how/where this research field could benefit with further research. From a broad perspective, further work is required to delineate the precise mechanisms through which obesity exacerbates the age-related loss of muscle mass and function to better understand the cause(s) and treatments of sarcopenic-obesity. For example, there is a need to understand the specific role of myosteatosis, lipid species and metabolites, in age-related muscle anabolic resistance, particularly in obese individuals. Indeed, further exploration of the role of intramuscular lipids in obesity-induced muscle anabolic resistance and sarcopenia is pivotal, particularly with regard to the co-localization of lipid droplets and mitochondria. In addition, it is plausible to suggest that the MPS response to weight-bearing activity could be greater in obese vs. lean older individuals, due to the larger mechanical stimulus. This could provide some explanation for the compensation for the blunted MPS response to amino acids, despite an apparent better preservation of muscle mass in obese older individuals. Future studies should also attempt longitudinal observations of obesity-induced changes to skeletal muscle as well as the effects of lifelong high calorie dietary patterns and sedentarism, and the reversal of obesity on skeletal muscle to truly understand the complex interactions between aging, obesity, skeletal muscle mass regulation and metabolic health parameters. Finally, as reduced physical activity is typically an inherent characteristic of obesity, further research is required to delineate the mechanisms associated with obesity and physical activity, independently, to the age-associated deterioration of skeletal muscle, as it is often difficult to distinguish between the two.

### Conclusion

The prevalence of sarcopenia, obesity, and sarcopenic-obesity are increasing globally, posing a considerable challenge to public health, healthcare resources, and the global economy. Sarcopenic-obesity is associated with accelerated functional decline, chronic disease risk, and increased risk of mortality. In contrast, increased levels of muscle mass, irrespective of adiposity, are associated with reduced risk of mortality. Despite evidence reporting greater amounts of muscle mass, but not muscle quality, in obese vs. normal weight older individuals in support of an “age-associated obesity paradox,” the current available data are inconclusive. Instead, obesity may impair muscle anabolic processes, potentially through compromised muscle quality and/or elevated levels of adipose-derived inflammatory cytokines. When combined with the age-related loss of muscle mass, this may further exacerbate the loss of muscle function in aging as well as the development of metabolic disease and disability. Older individuals are also likely disproportionately impacted by obesity at a functional level compared with younger obese individuals. The loss of muscle quality likely represents an important contributor to the impairment in physical function in older age. Indeed, intramyocellular lipid accumulation, a common characteristic of obesity, may interfere with contractile force production. Currently, however, the mechanisms through which obesity in older age may protect muscle quantity, yet seemingly impair muscle quality and function are unclear and warrant further investigation, as the complex interplay between obesity and sarcopenia makes it difficult to determine precise cause and effect.

## Author Contributions

PM completed the literature searches, review, and drafted the manuscript. PM, BS, and LB reviewed, edited, and approved the manuscript for its intellectual content. All authors contributed to the article and approved the submitted version.

## Conflict of Interest

The authors declare that the research was conducted in the absence of any commercial or financial relationships that could be construed as a potential conflict of interest.
